# Genetic Diversity and Population Structure of Head Blight Disease Causing Fungus *Fusarium graminearum* in Northern Wheat Belt of India

**DOI:** 10.3390/jof8080820

**Published:** 2022-08-05

**Authors:** Noyonika Kaul, Prem Lal Kashyap, Sudheer Kumar, Deepti Singh, Gyanendra Pratap Singh

**Affiliations:** 1 ICAR-Indian Institute of Wheat and Barley Research, Karnal 132001, Haryana, India; 2 Amity Institute of Microbial Technology, Amity University, Jaipur 303002, Rajasthan, India

**Keywords:** aggressiveness, head scab multi-locus sequence typing, mutation, phylogeny, population structure, recombination

## Abstract

Head blight or scab caused by *Fusarium graminearum* (FG), once ranked as a minor disease in wheat, is now emerging as one of the economically important diseases in India. The present study represents the first in-depth population genetic analysis of the FG from the northern wheat belt of India. In this study, multiple conserved gene sequences comprised of β-tubulin (TUB), translation elongation factor 1-α (TEF), and histone-3 (HIS) regions were used for multi-locus phylogenetic analysis of 123 geographically distinct *F. graminearum* isolates collected from four different states (Haryana (HR), Punjab (PB), Rajasthan (RJ) and West Bengal (WB)) of India. The phylogenetic and haplotype analysis showed the presence of thirty haplotypes in all the analyzed populations. The haplotypic diversity in the RJ population (Hd = 0.981) was higher than in the HR (Hd = 0.972), PB (Hd = 0.965) and WB population (Hd = 0.962). Recombination events (Rm = 12) and mutation events (485) were also detected. Analysis of molecular variance (AMOVA) indicated that genetic diversity was exclusively due to the differences within populations. The haplotype network was widely dispersed and not associated with specific populations, as a single common haplotype was not detected. The PB population contained both unique (H9, H10 and H11) and shared haplotypes (27 haplotypes) in a higher number in comparison to other geographical locations. Except for haplotype H22 (contains highly aggressive isolates), there was no specific linkage noticed between the isolate aggressiveness and haplotype. The concatenated sequences of all the three genes demonstrated a low level of genetic differentiation (Fst = −0.014 to 0.02) in the analyzed population. Positive values for the neutrality tests in PB, HR and RJ reveal a balancing selection mechanism behind the FG population structure. The WB population showed both positive and negative values of neutrality indices, indicating the role of both population expansion as well as balancing selection in structuring the FG population.

## 1. Introduction

**Figure 1:**
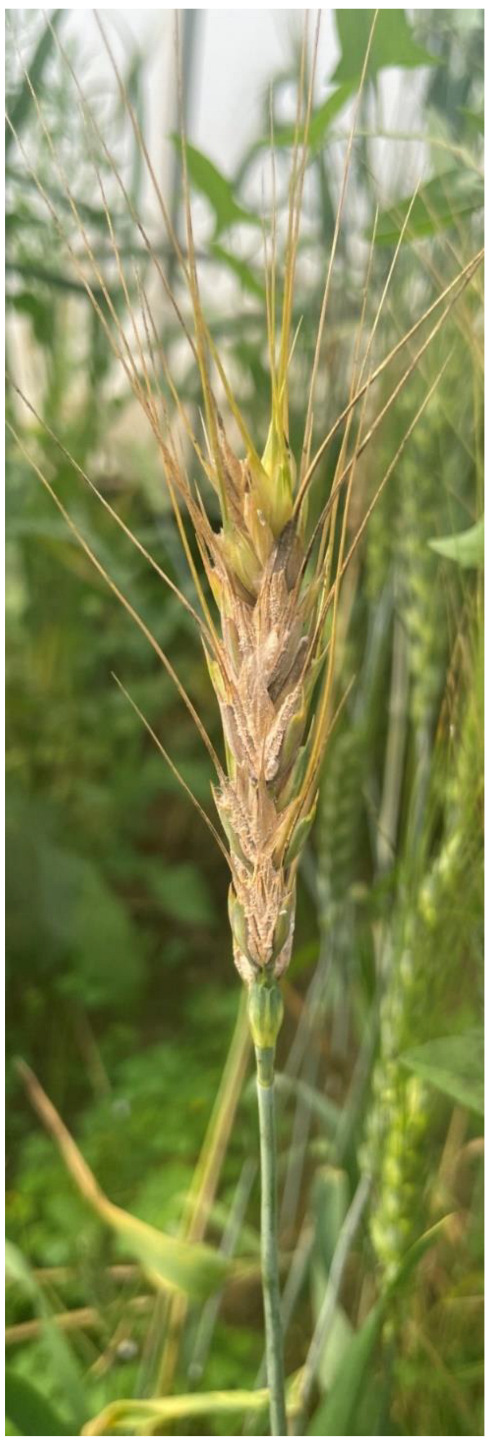
Typical symptoms of wheat head scab disease (*Fusarium graminareum*) on wheat spike.

*Fusarium* head blight (FHB) incited by *Fusarium graminearum* (FG) fungus is ranked as the one of the prime annihilating fungal diseases of wheat (*Triticum aestivum* L.) globally [1]. The published literature indicated that this disease drastically reduces the crop yield, leading to huge economic losses [2,3,4]. It has been noticed that yield losses are primarily linked with poor quality seed production. The contamination of infected seed grains with mycotoxins has been observed [5]. In India, the typical symptoms produced by the fungus appear majorly on the glumes and rachis of wheat plants in the form of water-soaked lesions. Later on, the fungus spreads within the wheat ear heads, resulting in the partial bleaching to complete blighting of attacked ear heads (Figure 1). Historically, the disease was first noticed in India in the Siang District of Arunachal Pradesh in the year 1974 [6]. Later on, the disease has been reported by other workers from Wellington [7] and Gurdaspur (Punjab, India) [8]. Bagga et al. [9] documented that the disease heavily attacked the wheat cultivar PDW 274 in Dera Baba Nanak region of Gurdaspur in Punjab district of India and resulted in noteworthy yield reduction. In addition to India, chronic appearance of the disease has been observed in different corners of the world and major regions including China, Brazil, USA, Canada, the former USSR, Eastern and Western Europe, Romania, etc. which account for more than 50% of global production [10,11]. In recent reports, it has been cautioned that *Fusarium* head blight is liable to enhance under reduced tillage-based wheat cultivation and further aggravated with climate shift especially in the northern part of India, which is recognized as the main wheat basket of India [4].

FG showed a broad host range, and it has the ability to infect different plants such as maize, sorghum, millets, rye, triticale, oats, etc. [12]. Various research evidence indicated that FHB disease is highly prone to humid to semi-humid areas of the world, especially where heavy and frequent rainfall with a high level of moisture exists in the atmosphere throughout the wheat cultivation season [13,14]. Unfortunately, such type of weather is prevalent in the northern part of India, especially during and after the anthesis stage of wheat, which directly affects the crop yield [4]. It has been observed that the fungus in the off-season survive in infected wheat straw in different grasses of the wheat family origin and in crop residues that remain in the soil after wheat harvest [15,16]. The incidence and severity of the FHB disease is determined by numerous factors such as quantity of airborne inocula (both inside and outside of the field), and the prevalence of humidity during and after the anthesis period [14,17]. Currently, fungicide (e.g., Tebuconazole, Triazoles, Prochloraz, etc.) sprays are important methods to preclude and conquer FHB disease in a short time and lessen *Fusarium* toxins production, despite agro-ecological and resistance development problems [2,18,19,20]. Hence, the deployment of FHB-resistant varieties is a sustainable, cost-effective, and environmentally friendly approach to FHB management. Unfortunately, the majority of the popular varieties cultivated in India are prone to FHB disease [4]. Moreover, the effectiveness of resistant varieties and implementation of different methods singly or in an integrated manner for the control of *Fusarium* spp. still exist as a daunting task because of the complex nature of FG and fungicide-resistant development in natural FG populations [21]. Therefore, an appraisal of genetic diversity and population structure of FG becomes mandatory to understand its evolutionary relationships with respect to environmental change, selection pressures, and other forces (e.g., mutation, genetic drift, gene flow, etc.) linked with evolutionary change [22,23]. Different research groups also advocated the essentiality of genetic structure analysis to decode the modes of recombination and distribution of isolates within and among populations [24,25]. Most importantly, FHB-resistant genotypes could be judiciously deployed if the pathogen population is known. At present, limited information is available regarding the distribution, genetic diversity and population structure of the FG population in India.

Molecular marker-driven technologies play a significant role in species identification because of their potential usage in exploring the population structure and genetic diversity within the fungal species and their isolates [26,27,28,29,30,31,32,33,34]. It is worth noting that high-throughput sequencing of amplicon markers from conserved genomic regions has provided new opportunities to decipher *Fusarium* diversity in agricultural crops in recent times. The molecular identification of fungi is primarily based on the internal transcribed spacer region (ITS) [35,36]. However, it has been observed that a number of species of *Fusarium* genus comprise orthologous regions and as a consequence, ITS region-based identification resulted in erratic and unreliable results. In this context, genomic regions representing translocation elongation factor 1-α (TEF), β-tubulin (TUB), histones (HIS) and calmodulin (CAL) have been widely used by different group of researchers to distinguish *Fusarium* spp. [33,37,38,39,40,41,42,43,44,45,46]. Yli-Mattila et al. [47] demonstrated the application if ITS, IGS, mtSSU, and TUB genomic region comparisons predicting the variation of *Fusarium* spp. Similarly, TEF-1α, β-tubulin and histone 3 regions have been explored by Webb et al. [48] and Taha et al. [49] for dissecting the genetic variability among *F. oxysporum* isolates. Recently, Fulton et al. [50] employed two different housekeeping genes (BT and TEF) for analyzing the phylogenetic kinship of *F. oxysporum* f. sp. *niveum* isolates from the major watermelon-producing regions in north, central, and south Florida. A flooding of reports are available that highlight the significance of TUB, TEF and HIS in resolving boundaries of fungal species and further revealed species identification and discrimination based on the combined sequences of gene loci as a more valuable genomic resource than single gene loci [51,52]. Unfortunately, not a single report regarding the dissection of genetic variation and population structure of FG in the major wheat-growing belt of North India using combined housekeeping gene sequences is available.

The history of FG in wheat in India is not too old, and therefore, the northern plains, a major wheat-growing belt of India, offers a paragon site to pinpoint the prime evolutionary mechanism acting on a newly developing FG isolate as it dispersed topically and regionally within the Indo-gangetic plains of India. Therefore, the current research was planned with an intention to obtain the answers to the question of whether FG contains distinct evolutionary lineages in the northern plains of India. The other questions include: (i) Is the phylogenetic and evolutionary structure of FG indicative of recombination or mutation?; and (ii) Is there a biogeographic relationship with the evolutionary structure of FG? The answers to these questions are essential to improve the understanding of the ecology of the plant pathogen and devise a better management strategy for the management of FHB disease in wheat.

## 2. Materials and Methods

### 2.1. Sampling and Pathogen Isolation

**Figure 2:**
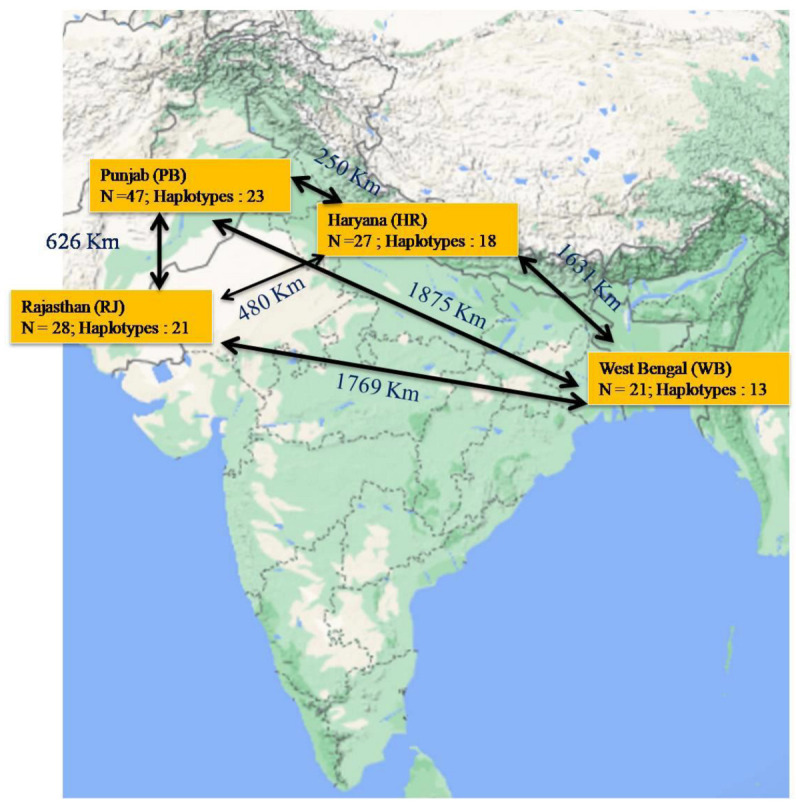
Map showing the sample collection sites in Northern wheat belt of India. N= Number of samples; Distance between two sample collecting states is mentioned over the black line.

One hundred and twenty three isolates of FG were used in the present study (Table 1). These isolates were collected during field surveys conducted from 2017 to 2022 in different wheat-growing fields in the four different states of India. These include: Punjab (PB; N = 47), Haryana (HR; N = 27), Rajasthan (RJ; N = 28) and West Bengal (WB; N = 21) (Figure 2). Disease wheat ear heads samples were showing typical premature bleaching with orange spore masses of the fungus on the infected spikelets and glumes (Figure 1). The symptomatic samples in the form of wheat ear heads were gathered in plastic bags and were taken into the laboratory. The detailed information of samples has been provided in Table 1. The isolation of the fungi was made by adopting the following procedure. Briefly, infected wheat samples were sliced into minute pieces of 2–3 mm and later surface-sterilized with ethanol (70%) for 30 s followed by NaOCl (1%) treatment for 1 min. After this, treated samples were washed twice with sterilized double-distilled water. After air drying, the treated wheat samples were placed on the Petri plates containing potato dextrose agar (PDA; Hi-Media India) and ampicillin (0.1 g 1^−1^). The Petri plates were incubated at 25 ± 2 °C. After five days of cultivation, the hyphal tip of fungus coming out from wheat tissue was placed onto other PDA-amended Petri plates and incubated at 25 ± 2 °C for conidia production. A single spore isolation methodology was adopted to raise the pure cultures of each FG isolate, which were stored at 4 °C as per the protocol of Kumar et al. [53].

### 2.2. Total Genomic DNA Extraction and Sequencing

Fungal isolates were grown for five days in shake cultures (200 rpm) at 25 ± 2 °C in 100 mL of potato dextrose broth (PDB; Himedia, India). The resulting mycelium was filtered through Whatman filter paper and thoroughly washed with distilled water. About 25–30 mg of mycelium was ground with the help of liquid nitrogen and used to extract the total genomic DNA as per the methodology of Kumar et al. [54]. The determination of total genomic DNA concentration was performed by using an Analytik Jena ScanDrop² instrument. Biometra Trios (Analytic Jena, Jena, Germany) machine, which along with the different primers mentioned in Table 2 were employed to amplify the fungal genomic DNA. The PCR master mix (25 μL) used in the thermal cycler comprised the following components: Go Taq Green master mix (12.5 μL; Promega Biotech India Pvt. Ltd. Jasola, New Delhi, India), fungal DNA (1 μL of 50 ng μL^−1^ concentration) and each primer pair (1 μL of 10 μM concentraion). In addition, nuclear-free water was used to make the total PCR master mix volume at 25 µL. The detailed information of the temperature profile used in the PCR for amplification of target genes (TUB, TEF and HIS) has been depicted in Table 2. Agarose gel electrophoresis was performed by using an E-BOX gel documentation system to see the PCR amplified amplicons of ≈500 bp ≈ 700 bp, and ≈350 bp fragments for TUB, TEF and HIS gene loci, respectively. The generated amplicons by each set of primers were freeze dried and sent to DNA sequencing analysis to Eurofins Genomics sequencing services, India. The obtained sequences of FG isolates were matched with *F. graminareum* isolates in National Center for Biotechnology Information (NCBI; https://www.ncbi.nlm.nih.gov/ (accessed on 11 June, 2021)), and we obtained the gene accession numbers.

### 2.3. Phylogenetic and Network Analysis

Nucleotide sequences of each gene loci (TUB, TEF and HIS) were matched with the gene sequences of respective loci available in the NCBI databank by basic local alignment search tool (BLAST; http://blast.ncbi.nlm.nih.gov/Blast.cgi (accessed on 11 June 2021)). Editing of gene sequences was conducted by using BioEdit 7.4.0.1 software [57]. ClustalW, a multiple sequence alignment program, was used for gene sequence alignment [58]. The DnaSP version 5 bioinformatic tool [59] was used to calculate the numbers of variable sites, parsimony informative sites, haplotypes and haplotype diversity. During the analysis, all positions containing gaps were removed. The DnaSP version 5 tool was also employed to determine the partitioning between populations from different studied sites and pair-wise comparisons of the nearest neighbor statistic, Snn [60] were calculated with 1000 permutations. Combined data sets of all three genes were employed to build a phylogenetic tree with the help of MEGA7 software [61], and the bootstrap value was adjusted at 1000 replications. A median joining haplotype network [62] was constructed for each of the four different populations based on different states and three population-based virulence features of isolates independently in PopART 1.7 [63]. The default epsilon value was fixed at zero. Analysis of molecular variance (AMOVA) was performed for each isolate independently in PopART 1.7 [63] to decipher the geographical grouping of genetic diversity. All the analyses were executed on a concatenated alignment of TUB, TEF and HIS data using PopART and were based on geographical locations and virulence features of the FG isolates. During the analysis, an experimental run was performed using complete deletion parameters.

### 2.4. Isolate Aggressiveness Analysis

Aggressiveness analysis of *F. graminearum* isolates was studied by inoculating susceptible wheat cultivar (cv. PBW343) with each isolate during 2021–2022 under a greenhouse. The mass production of FG isolates was completed by cultivating them on PDA media. After 15 days of incubation at 25 ± 2 °C, the inoculum was collected by rinsing the Petri plates with sterile distilled water containing Tween 20. Afterwards, the scraped mycelial mat with spores was passed through a double-layer sterile cheesecloth. Final spore inocolum concentration was adjusted at 5 × 10^6^ spore ml^−1^ with the help of a hemocytometer. The cotton wool ball technique [64] was used to inoculate FG isolates at the wheat anthesis stage. Five spikes per isolate were used. A perforated plastic bag was put over each of the inoculated wheat spike to avoid cross-contamination. Misting was performed to maintain the desired level of humidity (RH > 90%) in the greenhouse. Phenotypic disease data were collected by visual inspection of each inoculated wheat spike. The data pertaining to the healthy and infected spikes as well as infected spikelets per spike in each plant was taken at 15 days post inoculation. Disease severity (%) or percentage of infected spikelets were determined by following the below mentioned formula:

Disease severity (%) = [Total infected spikelets/Total spikelets per spike)] × 100.

The aggressiveness of all the FG isolates was categorized into four classes as highly aggressive (HA; FHB infection of more than 50%), moderately aggressive (MA; FHB infection ranged between 25–50%) and Least or weakly aggressive (LA; FHB infection below 25%).

## 3. Results

### 3.1. Molecular Identity Confirmation of F. graminareum Isolates

**Figure 3:**
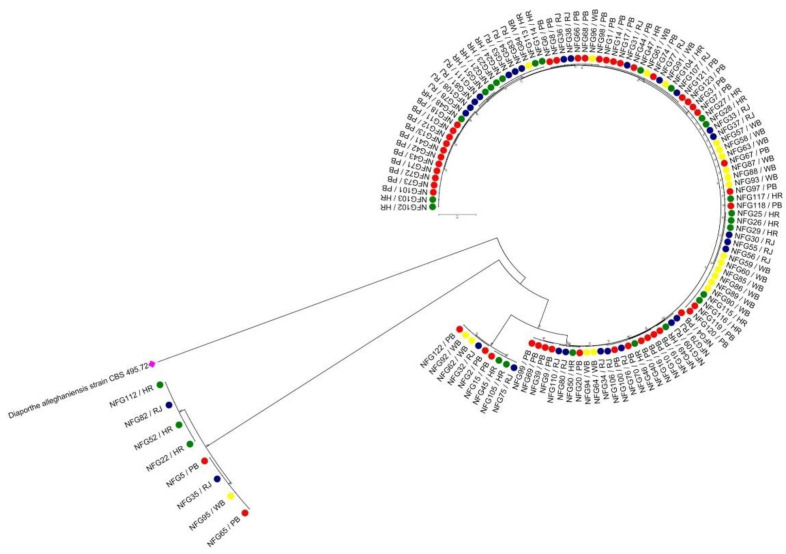
Phylogenetic relationship determined by using combined sequence of three gene loci (TUB, TEF and HIS sequences) and neighbor-joining (NJ) method. The percentage of replicate tree in the bootstrap test is 1000 replicates. The evolutionary distances were computed using the Kimura 2-parameter method. All positions containing gaps and missing data were eliminated. The tree was rooted with *Diaporthe alleghaniensis* strain CBS 495.72 [KC343733.1].

All the isolates based on the comparison of genomics regions for all the three loci (700 bp TEF, 500 bp TUB, and 350 bp HIS) confirmed their identity as *F. graminareum*. The sequences of all three genes of all 123 FG isolates reflected 99–100% similarity (Table 1). The sequences identified in this study have been deposited in GenBank, and the obtained accession numbers are mentioned in Table 1. After sequence alignment, the final combined dataset of TUB, TEF and HIS had 2045 characters (Table 3). The percentage of sequence similarity for FG isolates (N = 123) was also performed by making comparative analysis of the sequences of the FUSARIUM-ID database. The sequence similarity for FG isolates between 98.3 and 100% was found to be within the threshold value documented by O’Donnell et al. [40]. In addition, the maximum likelihood phylogenetic analysis also displayed strong support for different lineage. It has been noticed that all the 123 FG isolates were grouped into two major clusters: Cluster I (115 isolates) and Cluster II (8 isolates) (Figure 3).

### 3.2. Haplotypic Diversity

**Figure 4:**
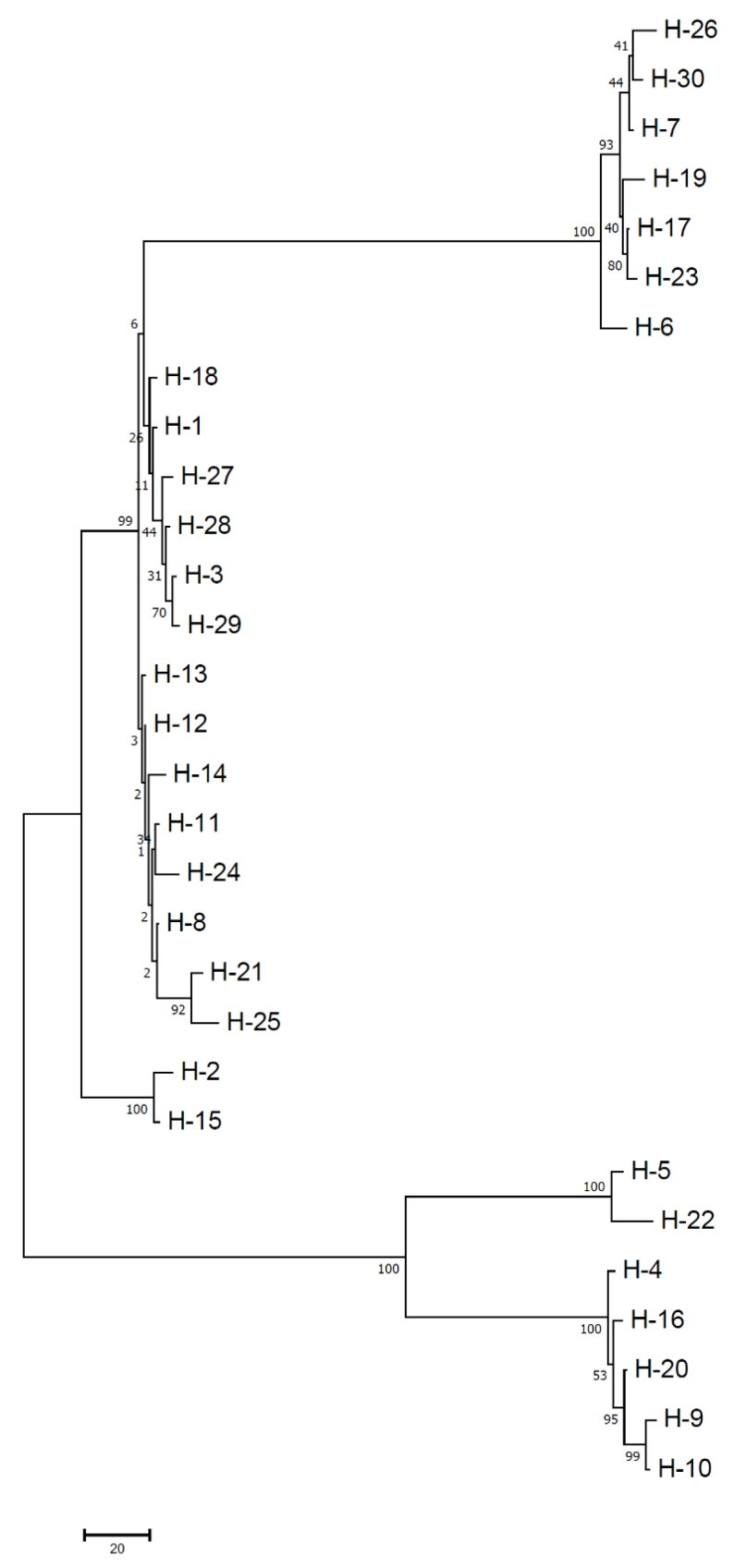
Unrooted maximum parsimony (MP) tree of haplotypes.

**Figure 5:**
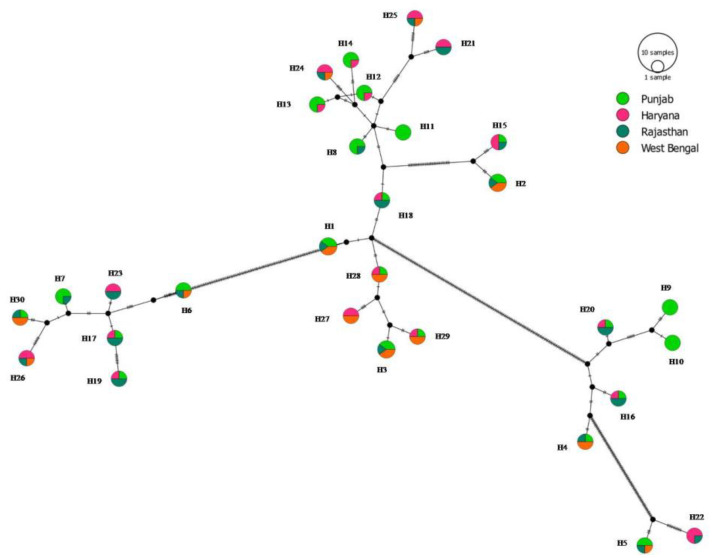
Median joining network of different haplotypes of FG population. Size of the circle is related with frequency of haplotypes. Colors indicate the proportion of individuals sampled in different populations within the study area.

The analysis of the combined gene loci reflected that both haplotype and nucleotide diversity were high (Hd = 0.974 and π = 0.083) (Table 4). The haplotype diversity ranged from 0.962 (WB) to 0.981 (RJ) (Table 4). A consensus maximum parsimony (MP) tree was generated for all the haplotype sequences observed in the study and shown in Figure 4. It is important to mention that MP analysis was preferred over ML owing to the fact that the TCS haplotype network was also based on parsimony-based statistics and hence will be a better option to make comparisons of groupings with strong bootstrap support. Figure 4 showed >90% bootstrap score and supported haplotype positioning with reference to distinct groupings of FG sequences. It has been observed that the network is reticulate type at the interior and star-like at the tip. Moreover, there was an absence of clearly evident centrally located single haplotype, from where various haplotypes came out (Figure 5). Isolates collected from the PB region were partitioned in 22 haplotypes, which is approximately 73.33% of the total haplotypes observed in the total analyzed population of the north plains of India. There was some evidence of geographic structure in the distribution of haplotypes. Punjab had a majority of the unique (H9, H10 and H11) and shared haplotypes (27 haplotypes) among all the studied populations. The predominant haplotypes were identified as H1, H2 and H3 and were observed in PB, RJ and WB. Sequences of isolates from PB belonged exclusively to three haplotypes (H9, H10 and H11 and absent in other populations. Interestingly, PB, RJ and WB shared seven haplotypes (H1–H6 and H30), while PB, HR and RJ shared three haplotypes (H15, H16, H17, H18, H19, and H20). Similarly, HR, RJ and WB shared three haplotypes (H24, H26 and H26). The population haplotype network for the 123 FG isolates was performed based on combined gene loci (Figure 5).

The haplotypes calculated on the basis of individual locus, i.e., TUB, TEF, and HIS, were 11, 15, and 17, respectively (Table 4). There were a total of 30 haplotypes observed in all the 123 FG isolates. Haplotype frequency was varies from four to five, where the majority of the haplotypes were composed of at least four isolates (Table 5). Haplotypes with higher frequencies were observed in PB from where the maximum number of isolates (23 haplotypes out of 47 isolates) was collected (Table 4). H10 and H11 had the maximum frequency (4) in the PB population (N = 47). Similarly, the RJ population shows a maximum frequency of six haplotypes (H16–H20 and H22). The frequency of H22 was found to be maximum in the HR population and included one isolate (NFG82) which belonged to the RJ population. WB populations have a maximum frequency of seven haplotypes (H1–H4, H27–H29 and H30) (Table 5). A single common haplotype was absent in all the analyzed populations. However, PB populations show some unique haplotypes (H9, H10 and H11) that were absent in other analyzed populations. The structure of the haplotype network matched with the MP topological tree.

### 3.3. Nucleotide Diversity

The nucleotide diversity among the four FG populations was low and lies between 0.070 (WB population) and 0.098 (RJ) (Table 4). The pair-wise genetic distances between different FG populations are shown in Table 6. The pairwise Fst for different populations is shown in Table 6. All the analyzed populations showed low Fst values (−0.012 to 0.025; *p* > 0.05). Negative Fst values were observed for PB and HR and WB and HR with *p* > 0.05 (Table 6). The results also indicated low nucleotide diversity for all the populations, i.e., WB (π = 0.070), PB (π = 0.072), RJ (π = 0.098), and HR (π =0.097) (Table 6).

### 3.4. Gene Flow and Genetic Divergence

The results (Table 7) indicated that Nm values for the RJ and PB (Nm = 88.51), RJ and HR (Nm = 61.87) and RJ and WB (Nm = 11.89) populations were more than 4. The genetic differentiation fixation coefficient was found to be non-significant among all the analyzed populations (Table 7). Snn values (Table 8) were found to be non- significant. When all the three loci were concatenated into a single sequence, a total of 12 recombination events were detected (Table 8).

### 3.5. Neutrality Test and Demographic History

The results of neutrality tests (Table 7) reflected non-significant negative values for both Tajima’s D and Fu’s FS statistic, indicating toward the spread of the FG population with supernumerary alleles. Furthermore, it has been noticed that the null hypothesis of neutrality for the combined gene loci based inference of FG populations was not jilted due to the existence of non-significant values of Tajima’s D and Fu’s FS. In addition, the analysis of the demographic population history performed on the basis of neutrality statistics for all the four sets of populations revealed a non-significant negative Tajima’s D value, indicating the presence of low-frequency polymorphism in the analyzed populations.

### 3.6. Analysis of Molecular Variance (AMOVA)

The results of AMOVA analysis demonstrated a variance of 100.84 within the FG populations. However, a negative percentage of variations (−0.84%) was recorded among FG populations (Table 9). The fixation indices (FI) among the populations were negative (FI = −0.0084), indicating a lack of genetic differentiation among the populations. 

### 3.7. Aggressiveness of Isolates

**Figure 6:**
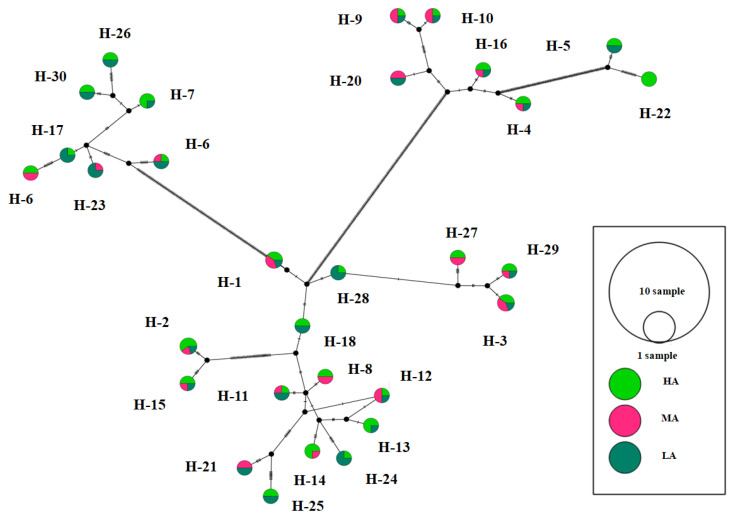
Median joining network according to different categories of aggressiveness of FG haplotypes. Size of the circle is related with aggressivity of the haplotypes. Colors indicate the proportion of individuals depicting the same level of the study area. HA: Highly aggressive; MA: Moderately aggressive and LA: Least aggressive.

The FG isolates were evaluated for their aggressiveness on wheat cultivar (cv. PBW343), and results in terms of HA, MA and LA are mentioned in Table 1. The results indicated that the aggressiveness of FG isolates varies from 42.28% isolates as highly aggressive to least aggressive (34.15%) and moderately aggressive (23.58%) (Table 1; Figure 6). The network also revealed no significant correlation between the genetic variability of FG isolates and aggressiveness levels. Furthermore, haplotype network analysis does not show any significant correlation with the FG isolates except for H22 haplotypes, which contained HA isolates.

## 4. Discussion

FG is one of the emerging and economically important diseases affecting wheat in India [4,65]. Diversity analysis is essential to infer the population genetics of such an important disease for framing cost-effective management tactics. In this context, a comprehensive understanding of genetic variation of the Indian FG population becomes necessity. Conserved region-based DNA markers are reported as one of the potential tools for determining the genetic variation, speciation of fungal pathogens and inferring their ancestral background [35,66]. FG diversity analysis based on TEF, HIS and TUB genes has discovered the existence of different putative subspecies of *Fusarium* in the Asian and sub-Saharan Africa terrains [67]. As Indian FG populations have not been analyzed so far to determine their diversity and population structure, therefore, the current investigation presented for the first time effort to quantify genetic variability in FG in northern plains of India using three highly versatile molecular markers, i.e., TUB, TEF and HIS. In this study, attempts have been made to (i) determine the identity of FG infecting wheat in the northern plains of India; (ii) confirm the level of DNA polymorphism of TUB, TEF and HIS sequences; (iii) decode the haplotype diversity and their distribution; and (iv) work out the phylogenetic kinship between FG isolates of Indian origin.

Previous published literature indicated that TEF is highly recommended for species delineation and rejected ITS sequences in offering superior taxa resolution for several of the fungal genera (e.g., *Trichoderma* and *Fusarium*) of the ascomycetes group [31,40]. From earlier published reports, it is cleared that when the distance between different *Fusarium* spp. to the nearest neighbor fungal species was deduced on the basis of ITS regions, very low genetic distance values were obtained, which ultimately resulted in the inferior resolution and poor taxon placement in phylogenetic lineages [68,69]. It is important to mention that slow evolving genes, for instance, TEF and HIS, serve excellent options in inferring phylogeny-based relationships. Contrarily, more recent evolutionary and speciation events can be captured from the gene sequences (e.g., TUB) displaying high evolutionary rates. Therefore, all the three genes were satisfying all the basic requirements needed for the phylogenetic analysis, as the concatenated sequences were composed of both variable introns and conserved exons [70] and hence selected for the present research investigation. According to the results of the current study and others [40], it is crucial to ascertain the DNA polymorphism in the sequences to be employed for the identification of intraspecies kinships and inferring phylogenetic lineages because of their direct influence on the correct and precise identity with strong phylogenetic arrangement and signal strength. Additionally, its influence on genetic diversity indices such as haplotype diversity and their dispersion have been reported [40]. The low level of genetic diversity noticed for wheat-associated FG populations in the northern plains of India based on concatenated gene sequences is not a rare event, as similar observations regarding the existence of a low level of phylogenetic diversity for FG populations in wheat has been documented by Castellá and Cabañes [71].

The significance of evolutionary forces on the FG population has been studied by conducting neutrality tests, which provides evidence regarding the divergence of combined gene loci sequence data from neutrality statistics. Moreover, the significant positive Tajima’s D values highlighted that all the three gene loci are experiencing population bottlenecks in PB, HR and RJ, where the FG population appears to be uniform and only a few sequences are in a deciding role for the development of the nascent population. This indicates that FG biology and the colonization pattern could act as one important factor behind population bottlenecks. Contrarily, significant negative values of Tajima’s D and F statistics in case of the WB population showed a strong purifying selection. This observation has been matched with the earlier report of Zhao et al. [72], where strong purifying selection in accordance with their important biological roles in cellular processes in fungi has been noticed.

Geography and climate are two of the most important epidemic linked factors and strongly influence the prevalence and establishment of *Fusarium* spp. as pathogens in various types of plants [71,73]. Usually, in nature, a mixture of both old and new haplotypes in the form of living descendants in field populations exists. However, the relationship between the two types of haplotypes may be reticulate and non-bifurcated because of non-dichotomous historical events, etc. [74]. Similarly, in the present study, a minimum of recombination events based on the combined three gene loci has been observed in the FG populations. This clearly reflects the mechanism of intragenic recombination behind the genetic variability in the pathogen population in the regions. However, it is important to mention here that Ma et al. [75] also confirmed the significance of horizontal gene transfer in the evolution of *Fusarium* genomes. Thus, high-frequency haplotypes (H1, H2 and H3) have been present in the population for a long time and detected from PB, RJ and WB population dominantly. Contrarily, other seem to be less frequent haplotypes and indicate toward more recent mutation events. Moreover, the terminal haplotypes (e.g., H2, H3, H5, H13–H15, H21, H22, H24–H26, H29, and H30) located at the network tips in the present study indicate a holocene pedigree rather than old ancestry (H1, H18 and H28) as placed in the interiors of the haplotype network. Carbone and Kohn [76] reported that newer haplotypes generally show restricted geographical dispersion in comparison to ancestral haplotypes, which have a wide geographical distribution and show restricted gene flow. However, the current study indicated toward a reticulate network, where both ancestral and nascent haplotypes occupied an internal celestial point of the network. The pattern of haplotype network and haplotype distribution in the studied sites of the northern plains illustrate that the FG isolates were separated from other regional isolates by a series of mutational events rather than geographical location (e.g., PB, HR, RJ and WB). These points indicate toward the possibility of the operation of both direct and indirect factors in the distribution of FG fungus, which is seed-, soil- and air-borne in nature and further supported by the haplotype network. The possibility of human-driven transfer of fungi in contaminated plant material and the transfer of FG inocula belong to other regions such as PB (where the maximum number of haplotypes was observed) or vice versa, which is followed by host adaptation and panoramic spatiotemporal dispersion. It has been observed that a number of haplotypes displayed only a difference at one site in the present study when compared with their genetically closest haplotype. These findings clearly reflect the significance of mutation in developing haplotype diversity as observed by earlier researchers in case of other fungal crop plant pathogens [30,77,78].

Haplotype analysis provides important information on the existence of different types of haplotypes (h), their diversity (Hd) and frequency. Usually, Hd values varied between 0 and 1, which reflects zero to high level of haplotype diversity, respectively [79]. In the current investigation, Hd (0.962–0.981) values revealed high levels of genetic variability in all the states. PB reflected maximum diversity (i.e., 27 haplotypes from 47 FG isolates) followed by RJ (i.e., 21 haplotypes from 28 FG isolates). It is important to mention that several haplotypes have been shared among different populations that clearly hinted toward the significance of asexual reproduction and effective spore migration. Two other points may help to explain the observed level of high genetic variability among FG isolates. These include the possibility of the existence of multiple founder populations, which resulted in population admixture as well as dispersion due to the assemblage of different alleles in FG populations, as clearly extrapolated from mutation events (both cumulative and shared mutational events) recorded in the current research. This observation has been supported by the high level of population admixture noticed in network analysis in the current study. The results of the current study also pointed toward the mutation as an essential factor for the generation of diverse regional populations of FG that later resulted in the developments of a number of mutants from which new and virulent type isolates can arise. Moreover, sharing a number of haplotypes among FG populations supports multiple introductions of the identical haplotypes, which may be due to the asexual reproductive phase of FG. Overall, it seems that the analyzed FG populations are admixture ones and occupied haplotypes from different populations in the northern plains of India.

The population genetics analysis performed in the current study revealed that the FG population from RJ seems genetically similar with other populations of PB, HR and WB. This statement is clearly supported by the values of high gene flow (Nm = 11.89–88.51) and low genetic differentiation (Fst = 0.002−0.025). A similar observation of high gene flow among different subpopulations of *F. graminearum* in Canada and the USA has been documented [25,80]. Furthermore, the ANOVA analysis conducted in present study supports the previous statement, as a very high level of genetic variation (100%) among individuals within the population has been recorded. These observations further indicate toward the greater possibility of sexual reproduction in the regions and are well supported with earlier research findings related to the population genetic structure of FG populations from wheat in Canada and the USA [25]. There might be another plausible reason, i.e., infected wheat seed movement as a planting material among different regions as a prime cause of gene flow between isolates of distinct origins. However, the role of long-distance spore transfer of FG in determining the gene migration cannot be omitted because of the air-borne mode of dissemination of the fungus [81].

A high variation in the aggressiveness of FG isolates has been observed in FG isolates collections from different wheat-growing states. These results are in agreement with earlier workers’ findings in both within-field populations and crossing populations [82,83,84]. The study shows that highly aggressive isolates are widely distributed in the northern plains of India and reflects the robust genetic diversity among regional FG populations. However, an exhaustive understanding of localized FG populations and their potential to surmount disease management tactics is essential to secure the wheat farmers from this emerging disease threat in India.

## Figures and Tables

**Table 1:** FG isolates used in the current study.

Isolate	Region/State	Wheat Cultivar	Year	NCBI Gene Accession			Aggressiveness
				TUB	TEF	HIS	
NFG1	Punjab	PBW343	2018	OM169181	ON215826	ON215856	HA
NFG2	Punjab	HD2967	2018	OM169182	ON215827	ON215857	MA
NFG3	Punjab	HD2967	2018	OM169183	ON215828	ON215858	HA
NFG4	Punjab	PBW 550	2018	OM169184	ON215829	ON215859	HA
NFG5	Punjab	PBW343	2018	OM169185	ON215830	ON215860	LA
NFG6	Punjab	PBW752	2018	OM169186	ON215831	ON215861	LA
NFG7	Punjab	PBW502	2018	OM169187	ON215832	ON215862	HA
NFG8	Punjab	PBW502	2019	OM169188	ON215833	ON215863	MA
NFG9	Punjab	PBW343	2019	OM169189	ON215834	ON215864	MA
NFG10	Punjab	DBW187	2019	OM169190	ON215835	ON215865	MA
NFG11	Punjab	HD2967	2019	OM169191	ON215836	ON215866	LA
NFG12	Punjab	PBW343	2019	OM169192	ON215837	ON215867	HA
NFG13	Punjab	PBW 757	2019	OM169193	ON215838	ON215868	LA
NFG14	Punjab	PBW502	2019	OM169194	ON215839	ON215869	HA
NFG15	Punjab	HD2967	2019	OM169195	ON215840	ON215870	HA
NFG16	Punjab	DBW187	2019	OM169196	ON215841	ON215871	MA
NFG17	Punjab	DBW187	2019	OM169197	ON215842	ON215872	LA
NFG18	Punjab	PBW 757	2019	OM169198	ON215843	ON215873	LA
NFG19	Punjab	HD2967	2019	OM169199	ON215844	ON215874	MA
NFG20	Punjab	DBW187	2019	OM169200	ON215845	ON215875	MA
NFG21	Haryana	UP 2338	2019	OM169201	ON215846	ON215876	LA
NFG22	Haryana	HD2967	2019	OM169202	ON215847	ON215877	HA
NFG23	Haryana	DBW187	2019	OM169203	ON215848	ON215878	LA
NFG24	Haryana	HD2967	2019	OM169204	ON215849	ON215879	LA
NFG25	Haryana	HD2967	2019	OM169205	ON215850	ON215880	HA
NFG26	Haryana	DBW187	2019	OM169206	ON215851	ON215881	LA
NFG27	Haryana	DBW187	2019	OM169207	ON215852	ON215882	HA
NFG28	Haryana	HD2967	2019	OM169208	ON215853	ON215883	HA
NFG29	Haryana	HD2967	2019	OM169209	ON215854	ON215884	MA
NFG30	Rajasthan	HD 2824	2019	OM169210	ON215855	ON215885	HA
NFG31	Rajasthan	HD 2824	2019	ON215733	ON215979	ON215886	LA
NFG32	Rajasthan	DBW187	2019	ON215734	ON215980	ON215887	HA
NFG33	Rajasthan	HD 3118	2019	ON215735	ON215981	ON215888	MA
NFG34	Rajasthan	PBW343	2019	ON215736	ON215982	ON215889	HA
NFG35	Rajasthan	HD2967	2019	ON215737	ON215983	ON215890	HA
NFG36	Rajasthan	HD2967	2019	ON215738	ON215984	ON215891	HA
NFG37	Rajasthan	RAJ 4079	2020	ON215739	ON215985	ON215892	LA
NFG38	Rajasthan	HD 2824	2020	ON215740	ON215986	ON215893	HA
NFG39	Punjab	PBW502	2020	ON215741	ON215987	ON215894	LA
NFG40	Punjab	WB 2	2020	ON215742	ON215988	ON215895	LA
NFG41	Punjab	DBW187	2020	ON215743	ON215989	ON215896	HA
NFG42	Punjab	WB 2	2020	ON215744	ON215990	ON215897	MA
NFG43	Punjab	DBW187	2020	ON215745	ON215991	ON215898	HA
NFG44	Punjab	HD2967	2020	ON215746	ON215992	ON215899	HA
NFG45	Haryana	UP 2338	2021	ON215747	ON215993	ON215900	HA
NFG46	Haryana	UP 2338	2021	ON215748	ON215994	ON215901	LA
NFG47	Haryana	DBW303	2021	ON215749	ON215995	ON215902	HA
NFG48	Haryana	DBW303	2021	ON215750	ON215996	ON215903	HA
NFG49	Haryana	DBW187	2021	ON215751	ON215997	ON215904	HA
NFG50	Haryana	DBW187	2021	ON215752	ON215998	ON215905	MA
NFG51	Haryana	HD2967	2021	ON215753	ON215999	ON215906	MA
NFG52	Haryana	DBW303	2021	ON215754	ON216000	ON215907	HA
NFG53	Rajasthan	HD3086	2021	ON215755	ON216001	ON215908	LA
NFG54	Rajasthan	UP 2338	2021	ON215756	ON216002	ON215909	LA
NFG55	Rajasthan	PBW343	2021	ON215757	ON216003	ON215910	HA
NFG56	Rajasthan	HD3086	2021	ON215758	ON216004	ON215911	LA
NFG57	West Bengal	DBW187	2021	ON215759	ON216005	ON215912	HA
NFG58	West Bengal	Shatabadi	2021	ON215760	ON216006	ON215913	LA
NFG59	West Bengal	HD2967	2021	ON215761	ON216007	ON215914	HA
NFG60	West Bengal	Shatabadi	2021	ON215762	ON216008	ON215915	LA
NFG61	West Bengal	DBW187	2021	ON215763	ON216009	ON215916	HA
NFG62	West Bengal	Prodip	2021	ON215764	ON216010	ON215917	HA
NFG63	West Bengal	HD3086	2021	ON215765	ON216011	ON215918	HA
NFG64	West Bengal	Prodip	2021	ON215766	ON216012	ON215919	LA
NFG65	Punjab	DBW303	2021	ON215767	ON216013	ON215920	LA
NFG66	Punjab	DBW303	2021	ON215768	ON216014	ON215921	LA
NFG67	Punjab	PBW550	2021	ON215769	ON216015	ON215922	HA
NFG68	Punjab	PBW502	2021	ON215770	ON216016	ON215923	HA
NFG69	Punjab	DBW187	2021	ON215771	ON216017	ON215924	MA
NFG70	Punjab	PBW 757	2021	ON215772	ON216018	ON215925	HA
NFG71	Punjab	HD2967	2021	ON215773	ON216019	ON215926	MA
NFG72	Punjab	HD2967	2021	ON215774	ON216020	ON215927	LA
NFG73	Punjab	PBW550	2021	ON215775	ON216021	ON215928	HA
NFG74	Punjab	DBW222	2021	ON215776	ON216022	ON215929	HA
NFG75	Rajasthan	DBW187	2021	ON215777	ON216023	ON215930	MA
NFG76	Rajasthan	HD3086	2021	ON215778	ON216024	ON215931	HA
NFG77	Rajasthan	DBW187	2021	ON215779	ON216025	ON215932	LA
NFG78	Rajasthan	PBW343	2021	ON215780	ON216026	ON215933	HA
NFG79	Rajasthan	HD3086	2021	ON215781	ON216027	ON215934	MA
NFG80	Rajasthan	HD2967	2021	ON215782	ON216028	ON215935	LA
NFG81	Rajasthan	RAJ 4252	2021	ON215783	ON216029	ON215936	MA
NFG82	Rajasthan	HD 2864	2021	ON215784	ON216030	ON215937	HA
NFG83	Rajasthan	DBW222	2021	ON215785	ON216031	ON215938	MA
NFG84	West Bengal	HD2733	2022	ON215786	ON216032	ON215939	HA
NFG85	West Bengal	DBW187	2022	ON215787	ON216033	ON215940	LA
NFG86	West Bengal	DBW 107	2022	ON215788	ON216034	ON215941	HA
NFG87	West Bengal	DBW303	2022	ON215789	ON216035	ON215942	MA
NFG88	West Bengal	DBW187	2022	ON215790	ON216036	ON215943	LA
NFG89	West Bengal	DBW 107	2022	ON215791	ON216037	ON215944	HA
NFG90	West Bengal	HD3086	2022	ON215792	ON216038	ON215945	LA
NFG91	West Bengal	Shatabadi	2022	ON215793	ON216039	ON215946	MA
NFG92	West Bengal	HD2733	2022	ON215794	ON216040	ON215947	HA
NFG93	West Bengal	HD2967	2022	ON215795	ON216041	ON215948	MA
NFG94	West Bengal	DBW303	2022	ON215796	ON216042	ON215949	MA
NFG95	West Bengal	HD2967	2022	ON215797	ON216043	ON215950	HA
NFG96	West Bengal	DBW 107	2022	ON215798	ON216044	ON215951	MA
NFG97	Punjab	DBW187	2022	ON215799	ON216045	ON215952	HA
NFG98	Punjab	DBW222	2022	ON215800	ON216046	ON215953	MA
NFG99	Punjab	HD 3226	2022	ON215801	ON216047	ON215954	HA
NFG100	Punjab	HD3086	2022	ON215802	ON216048	ON215955	MA
NFG101	Punjab	DBW222	2022	ON215803	ON216049	ON215956	LA
NFG102	Haryana	HD3086	2022	ON215804	ON216050	ON215957	MA
NFG103	Haryana	DBW303	2022	ON215805	ON216051	ON215958	HA
NFG104	Haryana	DBW303	2022	ON215806	ON216052	ON215959	MA
NFG105	Haryana	DBW303	2022	ON215807	ON216053	ON215960	LA
NFG106	Rajasthan	DBW222	2022	ON215808	ON216054	ON215961	HA
NFG107	Rajasthan	DBW222	2022	ON215809	ON216055	ON215962	La
NFG108	Rajasthan	HD 2864	2022	ON215810	ON216056	ON215963	LA
NFG109	Rajasthan	PBW343	2022	ON215811	ON216057	ON215964	HA
NFG110	Rajasthan	HD 2824	2022	ON215812	ON216058	ON215965	LA
NFG111	Rajasthan	HD 2864	2022	ON215813	ON216059	ON215966	LA
NFG112	Haryana	DBW303	2022	ON215814	ON216060	ON215967	HA
NFG113	Haryana	DBW303	2022	ON215815	ON216061	ON215968	LA
NFG114	Haryana	KRL210	2022	ON215816	ON216062	ON215969	LA
NFG115	Haryana	DBW187	2022	ON215817	ON216063	ON215970	LA
NFG116	Haryana	DBW222	2022	ON215818	ON216064	ON215971	HA
NFG117	Haryana	HD 3226	2022	ON215819	ON216065	ON215972	MA
NFG118	Punjab	HD2967	2022	ON215820	ON216066	ON215973	LA
NFG119	Punjab	HD3086	2022	ON215821	ON216067	ON215974	LA
NFG120	Punjab	DBW222	2022	ON215822	ON216068	ON215975	HA
NFG121	Punjab	DBW222	2022	ON215823	ON216069	ON215976	MA
NFG122	Punjab	HD 3226	2022	ON215824	ON216070	ON215977	LA
NFG123	Punjab	DBW303	2022	ON215825	ON216071	ON215978	LA

HA: Highly aggressive; MA: Moderately aggressive; LA: Least aggressive.

**Table 2:** Gene regions and primer pairs used in the current study.

Gene Region	Sequence (5′-3′)	Product Size (bp)	Optimized PCR Conditions	Reference
Translation elongation factor 1 alpha (TEF)	TEF1: ATGGGTAAGGAGGACAAGAC	≈700	95 °C: 5 min, (95 °C: 30 s, 56 °C: 30 s, 72 °C: 1 min) × 35 cycles 72 °C: 10 min	[55]
	TEF2: GGAAGTACCAGTGATCAT GTT			
Histone (HIS)	CYLH3F: AGGTCC ACTGGTGGCAAG	≈500	95 °C: 5 min, (95 °C: 30 s, 55 °C: 50 s, 72 °C: 1 min) × 35 cycles 72 °C: 5 min	[56]
	H3-1b: GCGGGCGAGCTGGATGTCCTT			
Beta-tubulin (TUB)	BT2a:GGTAACCAAATCGGTGCTGCTTTC		94 °C: 5 min, (94 °C: 30 s, 54 °C: 50 s, 72 °C: 1 min) × 35 cycles 72 °C: 10 min	[56]
	Bt2b: ACCCTCAGTGTAGTGACCCTTGGC	≈350		

**Table 3:** DNA polymorphism data for *F. graminearum* isolates based on tubulin (TUB), translation elongation factor (TEF) and histone (HIS) gene sequence comparisons.

Parameters	TUB	TEF	HIS	Combined
Number of sites	823	993	460	2045
Theta (per site) from Eta	0.012	0.120	0.003	0.083
Theta (per sequence) from Eta	1.857	38.995	1.300	90.059
Total number of mutations (Eta)	10.000	210.000	7.000	485.000
Fu and Li’s F *	1.333	1.184	1.703	2.718
Fu and Li’s D *	1.361	2.697	1.178	2.817
Average number of pairwise nucleotide differences, k	2.367	25.969	2.408	133.948
Total number of mutations, Eta	10	210	7	485
Minimum number of Recombination events, Rm	4	4	4	12
Tajima’s D	0.6814	−1.1005	1.93605	1.62305

* indicates neutrality statistics without an outgroup.

**Table 4:** DNA polymorphism data for *F. graminearum* isolates based on beta-tubulin (TUB), translation elongation factor 1 alpha (TEF) and histone (HIS) gene sequence comparisons.

Gene Loci	Region of Collection	Number of Isolates (N)	Number of Segregating Sites (S)	Number of Haplotypes (H)	Haplotype Diversity (Hd)	Nucleotide Diversity (π)
TUB	PB	47	5	7	0.816	0.013
	HR	27	6	10	0.900	0.019
	RJ	28	6	11	0.865	0.015
	WB	21	6	8	0.838	0.012
	Total	123	6	11	0.846	0.015
TEF	PB	47	197	12	0.891	0.056
	HR	27	195	11	0.883	0.128
	RJ	28	197	13	0.939	0.089
	WB	21	196	7	0.671	0.061
	Total	123	197	15	0.888	0.080
HIS	PB	47	7	14	0.920	0.006
	HR	27	7	10	0.855	0.005
	RJ	28	7	11	0.857	0.004
	WB	21	7	10	0.919	0.006
	Total	123	7	17	0.893	0.893
Combined loci	PB	47	384	23	0.965	0.072
	HR	27	381	18	0.972	0.097
	RJ	28	385	21	0.981	0.098
	WB	21	381	13	0.962	0.070
	Total	123	385	30	0.974	0.083

Haplotype diversity and nucleotide diversity are important indicators of population genetic variation. Haplotype and nucleotide diversities are generally considered to be low where haplotype diversity (Hd) and nucleotide diversity (π) are less than 0.5.

**Table 5:** Haplotype frequency (Freq) and distribution for *F. graminareum* isolates based on combined gene loci.

Haplotype	Frequency	Region (Isolates)
Hap_1	5	Punjab (NFG1, NFG121); Rajasthan (NFG31); West Bengal (NFG61, NFG91)
Hap_2	5	Punjab (NFG2 and NFG122); Rajasthan (NFG32); West Bengal (NFG62, NFG92)
Hap_3	5	Punjab (NFG3, NFG123); Rajasthan (NFG33); West Bengal (NFG63, NFG93)
Hap_4	4	Punjab (NFG4); Rajasthan (NFG34); West Bengal (NFG64, NFG94)
Hap_5	4	Punjab (NFG5, NFG65); Rajasthan (NFG35); West Bengal (NFG95)
Hap_6	4	Punjab (NFG6, NFG96); Rajasthan (NFG36); West Bengal NFG66,
Hap_7	4	Punjab (NFG7, NFG97 NFG67); Rajasthan (NFG37)
Hap_8	4	Punjab (NFG8, NFG98, NFG68); Rajasthan (NFG38)
Hap_9	4	Punjab (NFG9, NFG39, NFG69, NFG99)
Hap_10	4	Punjab (NFG10, NFG40, NFG70, NFG100)
Hap_11	4	Punjab (NFG11, NFG41, NFG71, NFG101)
Hap_12	4	Punjab (NFG12, NFG42, NFG72); Haryana (NFG102)
Hap_13	4	Punjab (NFG13, NFG43, NFG73); Haryana (NFG103)
Hap_14	4	Punjab (NFG14, NFG44, NFG74); Haryana (NFG104)
Hap_15	4	Punjab (NFG15); Haryana (NFG45, NFG105); Rajasthan (NFG75)
Hap_16	4	Punjab (NFG16); Haryana (NFG46); Rajasthan (NFG76, NFG106)
Hap_17	4	Punjab (NFG17); Haryana (NFG47); Rajasthan (NFG77, NFG107)
Hap_18	4	Punjab (NFG18); Haryana (NFG48); Rajasthan (NFG78, NFG108)
Hap_19	4	Punjab (NFG19); Haryana (NFG49); Rajasthan (NFG79, NFG109)
Hap_20	4	Punjab (NFG20); Haryana (NFG50); Rajasthan (NFG80, NFG110)
Hap_21	4	Haryana (NFG21, NFG51); Rajasthan (NFG81, NFG111)
Hap_22	4	Haryana (NFG22, NFG52, NFG112); Rajasthan (NFG82)
Hap_23	4	Haryana (NFG23, NFG113); Rajasthan (NFG53, NFG83)
Hap_24	4	Haryana (NFG24, NFG114), Rajasthan (NFG54);West Bengal (NFG84)
Hap_25	4	Haryana (NFG25 (NFG115); Rajasthan (NFG55); West Bengal (NFG85)
Hap_26	4	Haryana (NFG26 NFG116); Rajasthan (NFG56); West Bengal (NFG86)
Hap_27	4	Haryana (NFG27, NFG117); West Bengal (NFG57, NFG87)
Hap_28	4	Haryana (NFG28); West Bengal (NFG58, NFG88); Punjab (NFG118)
Hap_29	4	Punjab (NFG119); Haryana (NFG29); West Bengal (NFG59, NFG89)
Hap_30	4	Punjab (NFG120); Rajasthan (NFG30), West Bengal (NFG60, NFG90)

**Table 6:** Neutrality tests statistics observed in current study.

Test Method	PB		HR		RJ		WB	
	S *	*p*	S	*p*	S	*p*	S	*p*
Tajima’s D	0.809	*p* > 0.10	0.037	*p* > 0.10	0.614	*p* > 0.10	−0.978	*p* > 0.10
Fu and Li’s D	2.176	*p* < 0.02	1.857	*p* < 0.02	1.638	*p* < 0.02	0.356	*p* > 0.10
Fu and Li’s F	1.988	*p* < 0.02	1.486	*p* < 0.10	1.535	*p* < 0.10	−0.064	*p* > 0.10
Fu’s Fs	24.788		11.400		6.389		13.859	

S * represents statistics of Tajima’s D, Fu and Li’s D, Fu and Li’s F and Fu’s Fs; *p* < 0.05 indicates significant differences, rejecting the null hypothesis; *p* > 0.10 indicates no significant differences, following the neutrality model; PB: Punjab; HR: Haryana; RJ: Rajasthan; and WB: West Bengal.

**Table 7:** Pair-wise Fst (above diagonal) and Nm (below diagonal) of *F. graminareum* populations from four states of northern plains of India.

Population	PB	HR	RJ	WB
PB	NA	−0.012 *	0.009 *	−0.014 *
HR	−21.32	NA	0.002 *	−0.019 *
RJ	88.51	61.87	NA	0.025 *
WB	−16.92	−11.45	11.89	NA

Fst: Genetic differentiation coefficient. When Fst ranges from 0 to 0.05, the genetic differentiation is low (Rousset 1997); Nm > 4 indicates that gene flow is frequent between populations, while negative value indicates an absence of gene flow; * Not statistically significant with *p* > 0.05. N/A: Not applicable.

**Table 8:** Snn statistics of four different population of *F. graminareum* isolates of northern plains of India.

Population	PB	HR	RJ	WB
PB	-			
HR	0.699			
RJ	0.535	0.412		
WB	0.636	0.657	0.549	-

Rm indicates the minimum number of recombination events; Snn value represents how often the nearest neighbor sequences are found in the same locality.

**Table 9:** Hierarchical analysis of molecular variance (AMOVA) in FG populations.

Source of Variation	Degree of Freedom	Sum of Squares	Variance Component	Variation (%)	*p*-Value	Fixation Index (FI)
Among populations	3	29,143.70	−122.70	−0.84	0.61	−0.0084
Within populations	119	1,754,661.87	14,745.06	100.84		

Statistical significance calculated at *p* > 0.05; Negative values for variations among populations are regarded as zero. Genetic structure for *F. graminareum* population was not detected through AMOVA. Negative FI values can be inferred as no genetic differences between the populations compared.

## Data Availability

The data are contained within the article or gene sequences generated in this study were deposited in GenBank under the accession numbers listed in Table 1.
